# Ventilation characteristics of cardiopulmonary resuscitation manikins: a mixed-methods bench study

**DOI:** 10.1016/j.resplu.2026.101318

**Published:** 2026-04-07

**Authors:** David Purkarthofer, Michael Weldi, Gerald Trathnigg, Sebastian Billig

**Affiliations:** a Medical University of Graz, Neue Stiftingtalstraße 6, Graz 8010, Styria, Austria; b Medizinercorps Graz, Austrian Red Cross, Münzgrabenstraße 151, Graz 8010, Styria, Austria; c Department of Health Studies, FH JOANNEUM University of Applied Sciences, Alte Poststraße 149, Graz 8020, Styria, Austria; d Department of Anaesthesia and Intensive Care, IRCCS San Raffaele Scientific Institute, Milan, Italy; e Med-STA GmbH, Elisabethinergasse 14, Graz 8020, Austria; f Elisabethinen Hospital, Elisabethinergasse 14, Graz 8010, Austria; g Department of Anaesthesiology, Faculty of Medicine, RWTH Aachen University, Pauwelsstraße 30, 52074 Aachen, Germany

**Keywords:** CPR training, CPR manikin, Simulation, Ventilation, Manual ventilation, Bag-mask ventilation, Ventilation training, Ventilation feedback, Cardiopulmonary resuscitation (CPR)

## Abstract

**Background:**

Sufficient ventilation during cardiopulmonary resuscitation (CPR) is increasingly recognised as a critical determinant of patient outcomes. The effectiveness of ventilation training, however, likely depends on the anatomical and mechanical fidelity of CPR manikins. While chest compression mechanics of manikins have been studied extensively, ventilation-related anatomy, airflow pathways, and respiratory mechanics have not been evaluated systematically.

**Methods:**

This study analysed 10 adult CPR manikins from three manufacturers. Airway and lung replicas were documented using photography, video laryngoscopy, and bronchoscopy. Expiratory tidal volume and air leakage were measured during volume-controlled mechanical ventilation via facemask, supraglottic airway devices and endotracheal tube. Mechanics of the respiratory system were described by the peak and plateau airway pressures and respiratory compliance.

**Results:**

Manikins differed substantially in airway design and expiratory airflow design. Three distinct expiratory mechanisms were identified, of which only one permitted exhalation through the airway and thus quantification of expiratory tidal volumes. Air leakage varied across manikins and airway devices, including during ventilation via advanced airway devices. Respiratory mechanics showed marked variability, with peak inspiratory pressures ranging from 21 ± 1 to 56 ± 1 mbar and compliances from 7 ± 0 to 51 ± 4 mL/mbar. Several models exhibited substantial leakage via the gastric channel of the i-gel® supraglottic airway.

**Conclusion:**

CPR manikins exhibited substantial variability in ventilation-related anatomy, airflow pathways, leakage and respiratory mechanics. None of the assessed manikins consistently approximated human ventilation characteristics across airway devices, underscoring the need for local evaluation of manikin-airway device combinations to optimise ventilation fidelity in CPR training.

## Highlights

• Ten adult CPR manikins showed major variability in airway/lung replica design.
• Three expiratory flow designs were identified; only one allowed VTE quantification.
• Marked leakage was present across manikins, even with advanced airways.
• i-gel® gastric channel leakage occurred in several manikin–device combinations.

## Graphical abstract

**Figure:**
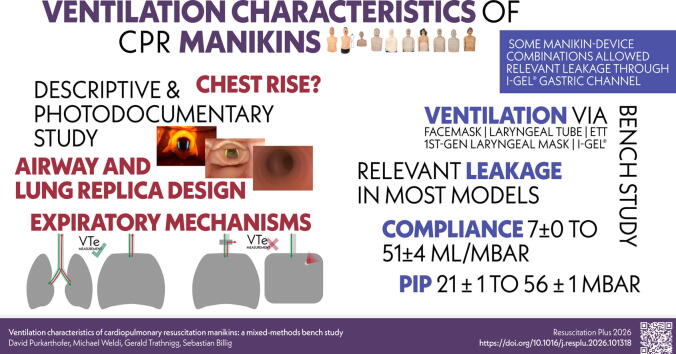

## Introduction

Ventilation during cardiopulmonary resuscitation (CPR) has received increasing attention in recent research, which has identified it as both a key determinant of patient outcomes and a technically demanding task for providers.^1^, ^2^ Especially bag-mask ventilation (BMV) is frequently inadequate, with real-world data demonstrating poor adherence to guideline-directed targets and an association with less favourable outcomes.^3^, ^4^, ^5^ This is mirrored by CPR training data: while training chest compressions has been a focus point, with sufficient data generated for current guidelines to advocate for the use of chest compression feedback during training,^6^ evidence guiding how to train ventilation remains limited. Notably, instructor feedback has been shown to be unreliable for judging alignment with guideline recommendations.^7^ Although ventilation feedback devices are available from different manufacturers and may improve care when used during cardiac arrest, it remains uncertain whether their use in training translates into later real-world performance gains.^8^, ^9^ Arguably, one kind of ventilation feedback has been there since manikins were used first: the haptic feedback on how much pressure is required to insufflate a certain volume during ventilation. While the mechanical characteristics of manikins regarding chest compressions were investigated as early as 1995,^10^, ^11^ this has not been done systematically for the ventilatory mechanical characteristics of CPR manikins.

Similar to chest compression training, mechanical fidelity to the human respiratory system is likely important for effective ventilation training.^12^, ^13^ Because most current ventilation feedback devices quantify only volume but not pressure,^14^, ^15^ realistic haptic feedback remains important even when these devices are used. Whereas chest compressions involve a single and simple interface (the manikin’s sternum), ventilation involves a more complex interface comprising the face, upper airway, lung replica, and thoracic cavity. Consequently, haptic feedback during ventilation may differ across airway management strategies.^16^, ^17^

In this study, we systematically described the design of the respiratory system replicas of a pragmatic sample of CPR manikins and measured the mechanical ventilation characteristics with standard airway adjuncts in a bench setup.

## Methods

We conducted a mixed-methods bench study. The study did not involve human participants or animals; thus ethical approval was not required.

### Descriptive assessment of airway and lung replica design

Airway and lung replicas were documented using photography (Canon EOS 5D Mark III, Tokyo, Japan), video laryngoscopy (VS-10 Medcaptain Medical Technology with M4 spatula, Guangdong, China) and endoscopy (Ambu aScope 4 Broncho Slim 3.8/1.2, Ambu A/S, Ballerup, Denmark). We assessed the presence and configuration of the oral cavity, oropharynx, hypopharynx, laryngeal inlet (laryngeal aditus) and trachea.

We also evaluated the presence of a visible chest rise, detectable expiratory flow, and any integrated ventilation feedback systems. Inspiratory and expiratory airflow pathways were documented photographically.

### Bench ventilation protocol

Following assessment of the airway design, ventilation mechanics were evaluated using a standard ventilator in volume-controlled ventilation mode (Dräger Savina; FiO_2_ 0.21; VT 500 mL; Ti 2s; RR 10/min; flow acceleration 50 mbar/s; *P*_max_ 60 mbar; PEEP 0 mbar) in line with contemporary guidelines.^18^ The tested airway interfaces included: (i) facemask (Disposable Facemask Adult S-L, Ambu A/S), (ii) laryngeal tube (LTS-D size 4–5, VBM Medizintechnik, Sulz a.N., Germany), (iii) first generation laryngeal mask (AuraOnce size 4–5, Ambu), (iv) second generation laryngeal mask (i-gel® size 4–5, Intersurgical, Sankt-Augustin, Germany) and (v) endotracheal tube (RüschR Super Safety Clear, size 7.5, Teleflex Wayne, PA 19087, USA).

Positioning of the face mask (FM), as well as insertion of airway devices, was performed by an anaesthesiology consultant. If fit or positioning was deemed suboptimal, adjustments were made to achieve the best attainable seal and lowest leakage. For facemask ventilation, both hands were used to optimise the seal, analogous to a two-person BMV technique. For supraglottic airway devices (SGA), common adult sizes were trialled, and results are reported for the best-fitting size. For second-generation laryngeal masks (i-gel® size 4–5) relevant leakage via the gastric channel was observed in some manikin–device combinations. Where present, measurements were repeated with the gastric channel open and occluded, and across sizes, and reported accordingly.

### Sampling and analysis

Two investigators with extensive experience in resuscitation education performed a pragmatic market sampling across low- to high-fidelity adult CPR manikins typically used for BLS/ALS training and assembled a convenience sample (Table 1). A manufacturer judged relevant (Gaumard Scientific, Miami, Florida, United States) could not be included because no representative was available in Austria at the time of the study.

**Table 1:** Overview of the analysed CPR manikins including descriptive information on the airway and lungs. Expiratory mechanism: Bellows-type lung with expiration through airway (I) or through one-way valve (II), or primitive replica with escape port (III). *Abbreviations:* BMV bag mask ventilation.

**Model**	**Manufacturer**	**BMV possible?**	**Realistic upper airway**	**Realistic lower airway**	**Chest rise on ventilation?**	**Expiratory flow present?**	**Expiratory mechanism**
AmbuMan SAM	Ambu	Yes	No	No	Yes	Yes	I
AmbuMan Wireless	Ambu	Yes	Yes	No	Yes	Yes	I
Mini Anne	Laerdal	Yes	No	No	Yes	No	III
Little Anne	Laerdal	Yes	No	No	Yes	No	III
Resusci Anne QCPR	Laerdal	Yes	Yes	No	Yes	No	II
Resusci Anne Advanced SkillTrainer	Laerdal	Yes	Yes	No	Yes	Yes	I
Sim Mom	Laerdal	Yes	Yes	Yes	Yes	Yes	I
Sim Man ALS	Laerdal	Yes	Yes	No	Yes	Yes	I
Nursing Anne Simulator	Laerdal	Yes	Yes	Yes	Yes	Yes	I
Brayden Advanced CPR Adult	Innosonian, Inc.	Yes	No	No	Yes	No	II

Ventilation data were sourced directly from the ventilator via a video camera capturing the measured data of the respirator and were subsequently manually extracted from the video. The manufacturer reports an accuracy of ±2 mbar for pressure estimates, ±10% for estimates of the expiratory tidal volume and ±35% for static compliance estimates.^19^ For each manikin-interface combination, 10 consecutive breaths equalling to one minute of ventilation were analysed. Supplement 1 provides an exemplary breath-by-breath visualisation for the Laerdal Resusci Anne Advanced SkillTrainer. All data were collected in a spreadsheet (Microsoft Excel version 16, Microsoft, Redmond, WA, USA). We report the following data: expiratory tidal volume (VT_E_); leakage, defined as $\frac{inspiratory\;minute\;volume-expiratory\;minute\;volume}{inspiratory\;minute\;volume}\times 100\%$; peak inspiratory pressure (PIP); plateau pressure (P_Plat_); and static compliance (C). Because leakage interferes with correct volume and pressure estimates,^20^ PIP, P_Plat_ and C are reported for the interface with the lowest leakage. Data are presented as the mean ± standard deviation (SD). Volume is reported in mL, pressure in mbar (convert to cmH_2_O by multiplying by ≈1.02), and compliance in mL/mbar (convert to mL/cmH_2_O by multiplying by ≈0.98).

## Results

Ten adult CPR manikins from three manufacturers were analysed for airway/lung replica design and ventilation mechanics (Table 1).

### Descriptive study

**Fig. 1:**
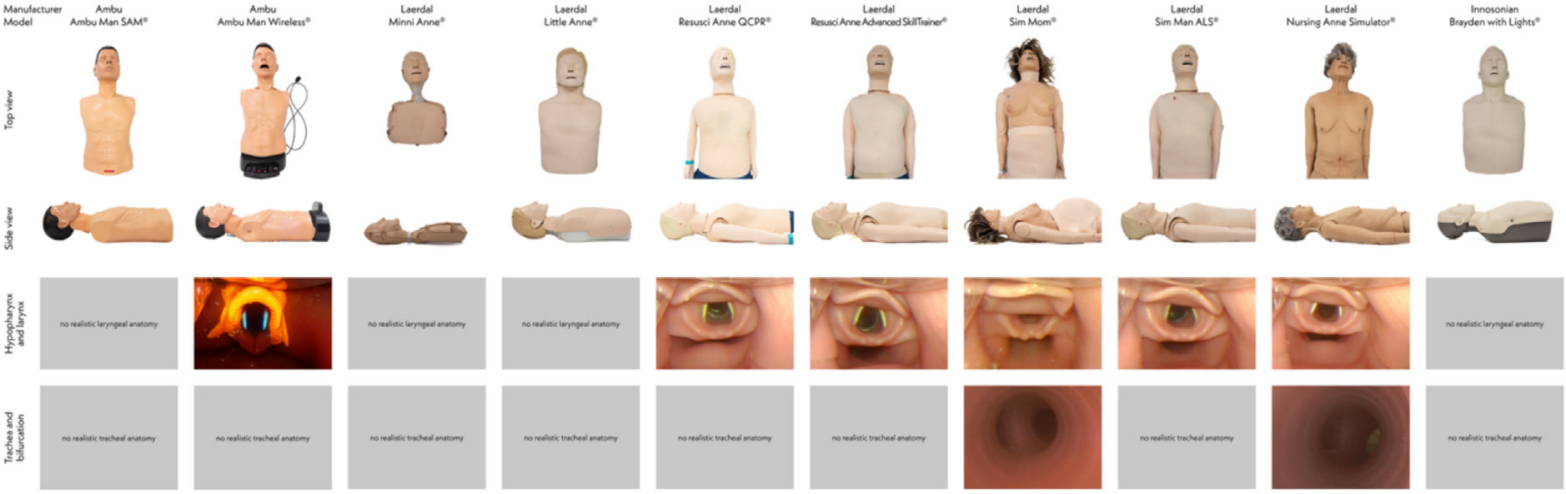
**Graphical overview of the analysed CPR manikins including top and lateral views and photos of hypopharynx, laryngeal inlet, and trachea**.

In all models with a simulated hypopharynx, laryngeal replicas (epiglottis and vocal cords) were present. However, only 2/10 models included a trachea and its main bifurcation (Laerdal Nursing Anne Simulator; Laerdal SimMom) (Fig. 1).

One of the evaluated models (Ambu Man Wireless) provided an analogue ventilation feedback system, whereas newer high-end manikins offered digital ventilation feedback via a dedicated application or a connected tablet.

While BMV was feasible and a chest rise was visible in all manikins, expiratory airflow at the airway was not consistently detectable (Table 1). We identified three distinct expiratory mechanisms (Fig. 3):

(i) Direct lung replica: the airway is connected to a bellows-type lung, allowing expiratory flow through the airway.
(ii) Directional-valve system: the airway connects to a lung replica via a one-way valve, with exhaled air diverted elsewhere rather than exiting through the airway.
(iii) Primitive lung replica with escape port: the airway is connected to a simplified lung chamber with an intentional constant leak, allowing air to escape through a small opening.

### Experimental bench study

#### Ventilatory properties

VT_E_ and leakage could only be quantified in manikins with expiratory mechanism type I, where exhalation occurred through the airway (Table 2). FM ventilation was feasible in all manikins with expiratory type I mechanisms and resulted in comparable air leakage with ventilation via supraglottic airway devices or an endotracheal tube. However, FM ventilation in the Nursing Anne Simulator with a latex face skin resulted in 100% leakage. Overall, air leakage varied by manikin model and airway device.

**Table 2:** Expiratory tidal volume (VT_E_) and percentual air loss per minute ventilation with face mask and standard airway devices. Only manikins with an airway design allowing expiration through the airway (type I expiratory mechanism) were included in this table. *Abbreviations:* FM facemask; LM laryngeal mask; LT laryngeal tube; ETT endotracheal tube.

**Model**	**Manufacturer**	**FM** **Size** **VT_E_ [mL]** **leakage**	**LM 1st gen** **Size** **VT_E_ [mL]** **leakage**	**LM 2nd gen** **Size (i-gel**®**)** **VT_E_ [mL]** **leakage**	**LT** **Size** **VT_E_ [mL]** **leakage**	**ETT** **Size** **VT_E_ [mL]** **leakage**
AmbuMan SAM	Ambu	M 391 ± 18 21%	n.a.	n.a.	n.a.	n.a.
AmbuMan Wireless	Ambu	L 363 ± 34 27%	5 388 ± 12 22%	4 388 ± 3 22%	4 425 ± 3 15%	7.5 372 ± 4 25%
Resusci Anne Advanced SkillTrainer	Laerdal	L 370 ± 34 26%	5 243 ± 22 52%	5 417 ± 16 16%	5 407 ± 14 17%	7.5 284 ± 17 42%
Sim Mom	Laerdal	L 133 ± 18 72%	5 59 ± 12 88%	4 294 ± 12 40%	5 300 ± 32 32%	7.5 190 ± 5 61%
Sim Man ALS	Laerdal	L 285 ± 18 42%	5 128 ± 9 74%	5 322 ± 18 35%	4 271 ± 19 45%	7.5 249 ± 16 50%
Nursing Anne Simulator	Laerdal	S/M/L 100%	4 28 ± 3 94%	4/5 100%	5 382 ± 9 23%	7.5 300 ± 9 39%

Respiratory mechanics showed a high degree of variability across the manikins (Table 3). PIP ranged from 21 ± 1 mbar (Laerdal SimMan ALS) to 56 ± 1 mbar (Laerdal SimMom), and compliance from 7 ± 0 mL/mbar (Laerdal SimMom) to 51 ± 4 mL/mbar (Laerdal Resusci Anne Advanced SkillTrainer).

**Table 3:** Respiratory mechanical properties of the analysed CPR manikins including peak inspiratory pressure (PIP), plateau pressure (P_Plat_) and compliance. Only manikins with an airway design allowing expiration through the airway (type I expiratory mechanism) were included in this table. 1 mbar ≈ 1.02 cmH_2_O; 1 mL/mbar ≈ 0.98 mL/cmH_2_O.

**Model**	**Manufacturer**	**PIP** **Mean ± SD [mbar]**	**P_Plat_** **Mean ± SD [mbar]**	**Compliance** **Mean ± SD [mL/mbar]**
AmbuMan SAM	Ambu	24 ± 0	11 ± 1	33 ± 2
AmbuMan Wireless	Ambu	25 ± 0	10 ± 0	50 ± 1
Resusci Anne Advanced SkillTrainer	Laerdal	32 ± 0	9 ± 1	51 ± 4
Sim Mom	Laerdal	56 ± 1	36 ± 2	7 ± 0
Sim Man ALS	Laerdal	21 ± 1	9 ± 0	40 ± 2
Nursing Anne Simulator	Laerdal	51 ± 0	39 ± 5	10 ± 2

#### Leakage via gastric channel of laryngeal mask

**Fig. 2:**
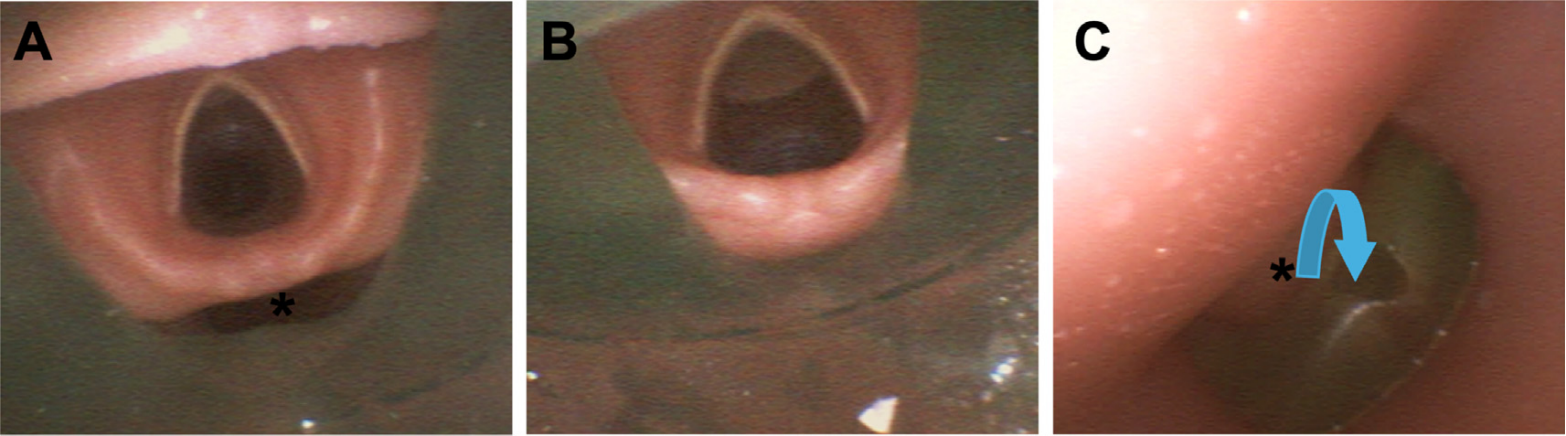
**Bronchoscopic images showing different sizes of the i-gel® laryngeal mask in relation to the larynx of a Laerdal SimMan ALS manikin**. (A) Larynx visualised via respiratory channel of a size 4 i-gel®. The asterisk (*) indicates the unsealed posterior larynx region. (B) Larynx via respiratory channel of a size 5 i-gel®. (C) Retrograde view of the 4 i-gel® via the oesophagus of the manikin. The asterisk corresponds to the unsealed posterior larynx region of image A. Air escapes from there via the gastric channel of the airway device resulting in significant leakage.

In some manikins, an air leak occurred during use of the i-gel® due to flow via the gastric channel, which decreased when the channel was manually occluded (Table 4). Endoscopy was used to identify the cause of this loss and revealed incomplete sealing around the laryngeal inlet with posterior leak pathways communicating with the gastric channel (Fig. 2).

**Table 4:** Manikins with observed air leakage via the gastric channel of the second-generation laryngeal mask (i-gel®). Only manikin-device-size combinations with differences between open and occluded gastric channel are included in this table. *Abbreviations:* VT_E_ Expiratory tidal volume.

**Model**	**Manufacturer**	**Device size**	**VT_E_ [mL] and leakage with open channel**	**VT_E_ [mL] and leakage with occluded channel**
AmbuMan Wireless	Ambu	4	0 ± 0 (100%)	388 ± 3 (22%)
		5	316 ± 4 (29%)	364 ± 7 (27%)
Resusci Anne Advanced SkillTrainer	Laerdal	4	171 ± 39 (66%)	259 ± 19 (48%)
		5	322 ± 24 (35%)	417 ± 16 (16%)
Sim Mom	Laerdal	4	207 ± 9 (57%)	294 ± 12 (40%)
Sim Man ALS	Laerdal	4	109 ± 0 (78%)	144 ± 18 (71%)

**Fig. 3:**
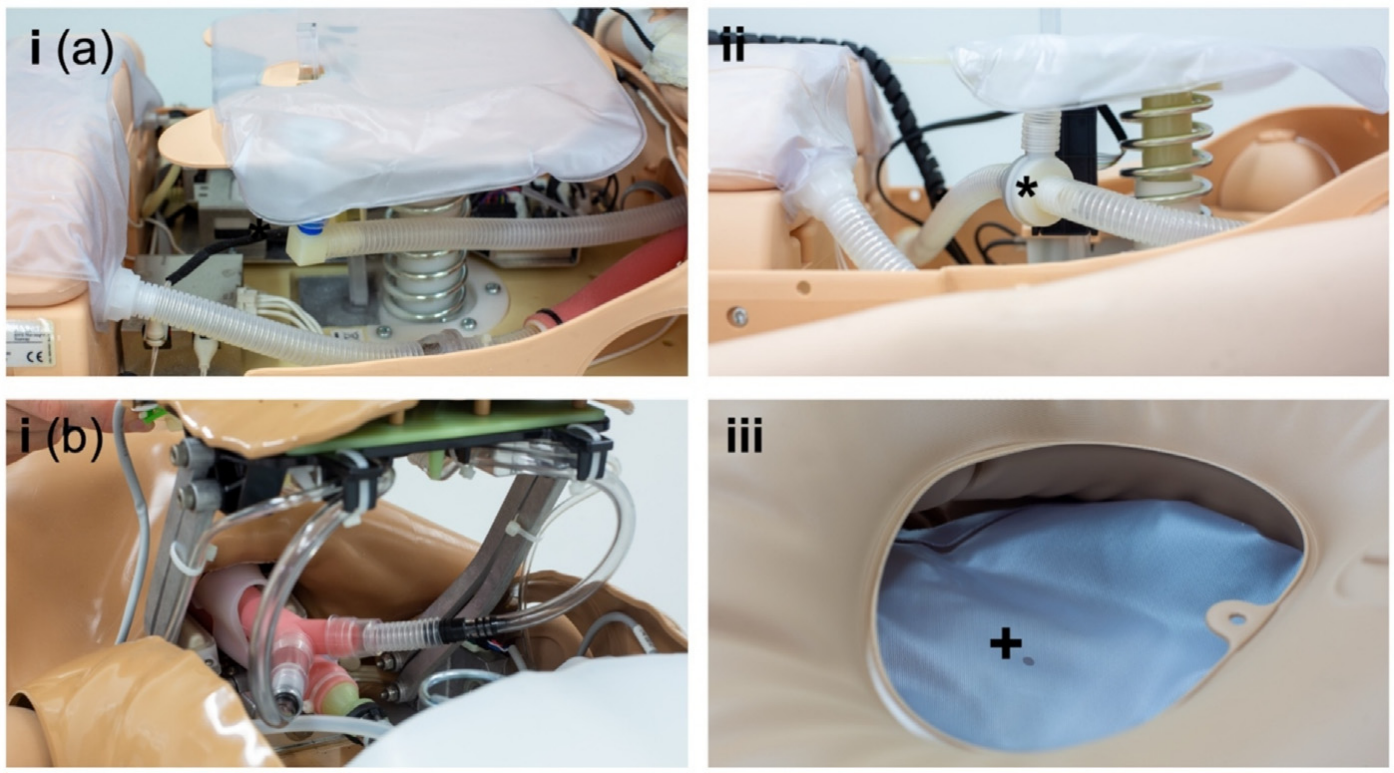
Expiratory mechanisms of the analysed manikins: (i) Airway directly connected to one (a) or two (b) bellow-type lungs. (ii) Airway connected to bellow-type lungs via directional valve (*), with expiratory flow being directed in a different direction. (iii) Airway connected to primitive replica with a small hole (+) as escape port.

## Discussion

In this experimental bench study assessing anatomic fidelity and respiratory mechanics across ten different CPR manikin models, we observed frequent leakage across manikin–airway device combinations, leakage through the gastric channel of the i-gel® in some models, and different expiratory mechanisms, only some of which permitted quantification of expiratory volume at the airway. These findings are directly relevant to CPR ventilation training and manikin-based research.

### Airway design

Consistent with prior work showing limited anatomical fidelity of manikin airways,^21^ we observed clinically relevant effects of the airway replica: even when a realistic airway was present that allowed for placement of SGA or an endotracheal tube, relevant air leakage could be observed in most manikin airway device combinations (Table 2). In some models, additional leakage was observed in the i-gel® via the gastric channel of the device. In these cases, the i-gel® sealed between the oro- and hypopharynx but did not seal around the laryngeal inlet as intended (Fig. 2), with airflow escaping via the gastric channel consequently. Flow through the gastric channel was observed in humans before,^22^ and interpreted as a warning sign for suboptimal position by the manufacturer.^23^ However, repositioning of the device, even with endoscopic guidance, to seal adequately was not possible in manikins where we observed this phenomenon. Given that the 2025 ERC guidelines newly recommend specifically i-gel® as a supraglottic airway device,^24^ educators should be aware that manikin-device interactions may introduce artefacts not necessarily representative of human anatomy.

### Airflow and leakage

**Fig. 4:**
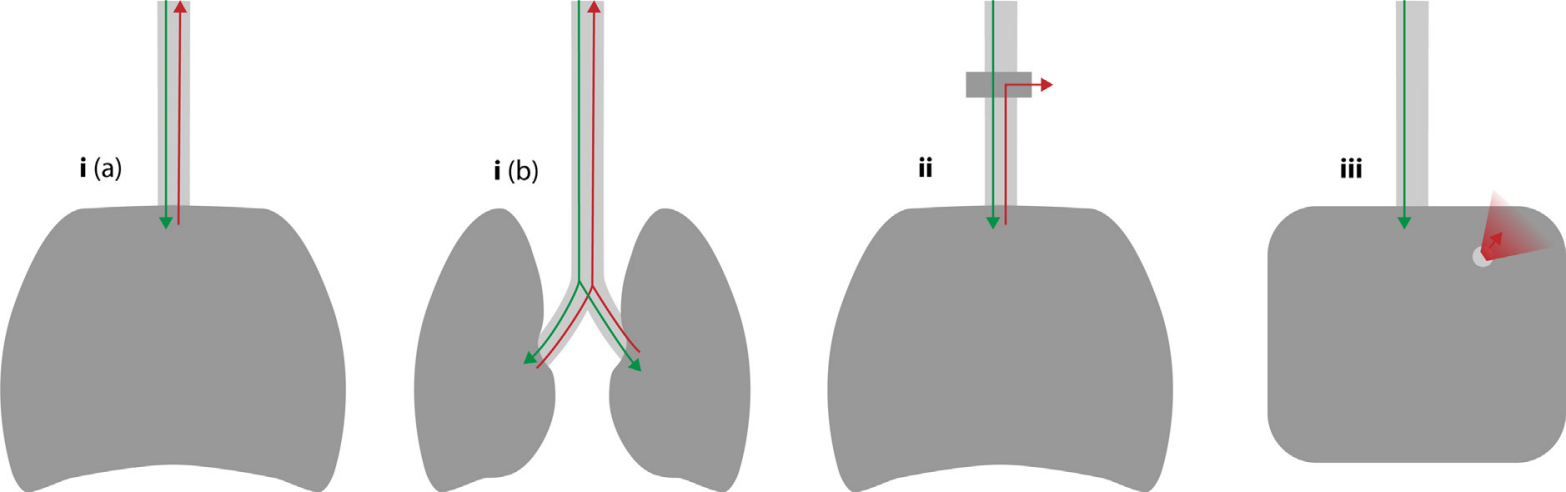
**Schematic depicting expiratory mechanisms of analysed manikins: (i) Airway directly connected to one (a) or two (b) bellow-type lungs. (ii) Airway connected to bellow-type lungs via directional valve, with expiratory flow being directed in a different direction. (iii) Airway connected to primitive replica with a small hole as escape port. The green arrow shows the inspiratory airflow, while the expiratory airflow is visualised in red**. (For interpretation of the references to colour in this figure legend, the reader is referred to the web version of this article.)

Two of the three expiratory mechanisms present in the manikins do not allow the calculation of leakage using the expiratory volume, as expiration does not happen through the airway (Figs. 3 and 4). These designs are commonly found in basic-life-support manikins intended for mouth-to-mouth ventilation, with the primary aim to reduce cross-contamination between trainees. At the same time, this precludes calculation of leakage from expiratory volume at the airway and limits the use of feedback systems that rely on expiratory flow.^25^

In our bench study, we observed measurable leakage in most manikin models, even when an endotracheal tube was in place. While some degree of leakage is recognised during CPR ventilation, especially via face mask,^4^ the magnitude and locations of leakage we identified suggest that manikin respiratory systems either permit airflow loss below the tracheal level or provide poorer sealing than in humans. Previous studies support this observation, reporting leakage exceeding 20% during mask ventilation in manikins,^26^ and reporting tidal volume discrepancies between mechanical ventilators and high-fidelity lung simulators when connected via manikin airways.^16^ The significant air leakage has two important implications for CPR training: first, it may distort haptic feedback during the inflation effort. Second, it may undermine the validity of ventilation feedback devices that rely on expiratory volume, potentially encouraging compensatory over-ventilation to ’reach the target’ – an adaptation that would not be appropriate when translated to human patients. This warrants close monitoring when ventilation feedback devices are used in training. Educators should be cautious in interpreting device-reported expiratory volumes and leakage unless the specific airway device–manikin combination has been validated for these measurements. Further research to quantify leakage during asynchronous ventilation with ongoing chest compressions is also warranted.

### Compliance

In most models, compliance at the target tidal volume fell within ranges reported in cardiac arrest patients, and porcine and cadaver models.^27^, ^28^ While we did not assess learner perception directly, these values suggest that haptic ‘stiffness’ during early CPR ventilation may be reasonably approximated in many manikins. Lower compliance due to CPR-associated lung oedema may develop later in resuscitation,^29^ but is less likely to dominate in the initial phase when 30:2 ventilation is typically used.

### Implications for CPR education and manikin-based research

Because our study evaluated ventilation in the absence of chest compressions, its findings are most directly transferable to 30:2 ventilation training, as used in basic life support and as an option in advanced life support when no advanced airway is in place, or when other circumstances make it preferable to asynchronous continuous ventilation. Physiologically, however, the pressure peaks and flow reversals arising from ventilation–compression interactions^30^ may, if similarly reproduced in manikins, contribute additional leakage beyond that observed in our experiments.

A practical mitigation, applicable across different settings, might be local validation of commonly used manikin–interface combinations using equipment capable of measuring flow and pressure, like modern mechanical ventilators or dedicated combined flow and pressure sensors.^31^ Such local validation can function as a training-technology validity check, aligning the selected manikin–airway device pair with the intended teaching or assessment use case. This would help identify models with extreme leakage or non-physiological mechanics and allow trainees to be briefed explicitly on expected differences between manikin feedback and patient ventilation. Because manikin-derived ventilation metrics may reflect simulator design and device interactions as much as learner performance, these measures should not be extrapolated to clinical ventilation quality without local validation of the simulator setup and performance thresholds.

Findings from this study may be transferable to other manikin platforms using identical or closely related airway modules, particularly within the Resusci Anne and SimMan product lines.

Future research could extend this work by characterising the effects of variability in tidal volume, flow, and pressure introduced by human-operated bag–mask ventilation or by assessing whether manikin ventilation fidelity in training translates to ventilation quality in clinical practice.

### Limitations

Our bench protocol does not address continuous asynchronous ventilation during chest compressions.

Although we were able to document that some manikins allow interaction between forces generated by chest compressions and the manikin’s lung replicas by placing them above or below the chest compression mechanism (Fig. 3), it was shown before that realistic simulation of this interaction requires a dedicated approach, not present in the manikins tested.^32^

Furthermore, the included manikin models may not be representative of all geographic regions and do not cover all manufacturers, which may limit generalisability. Only one unit per model was tested, so production variation, wear, and storage effects cannot be excluded. Finally, because interface placement was performed by an anaesthesiology consultant, operator-dependent effects may occur, particularly when placement is performed by less experienced clinicians.

## Conclusion

In conclusion, many CPR manikins approximate patient-like compliance during early CPR, but substantial non-physiological leakage and airflow pathway designs are common. Although some models reproduced selected aspects of ventilation, none of the assessed manikins consistently approximated human ventilation characteristics across airway devices. Certain manikin-device interactions, most notably leakage through the i-gel® gastric channel, may introduce non-physiological artefacts. Considering the prominent recommendation of the i-gel® in the 2025 ERC guidelines, these findings should encourage manufacturers to optimise their manikins’ airway modules. Given the diversity of models and airway devices, comprehensive testing of all combinations is impractical; we therefore advise caution when interpreting particularly expiratory tidal volumes and leakage, and recommend local evaluation of the manikins and airway devices in use to identify limitations and optimise ventilation training fidelity.

## Artificial intelligence use

The authors declare that generative AI (ChatGPT, OpenAI) was used to improve grammar and readability. No AI tool was used to generate scientific content. All suggested edits were reviewed by the authors, who take full responsibility for the final manuscript.

## CRediT authorship contribution statement

**David Purkarthofer:** Writing – review & editing, Writing – original draft, Visualization, Investigation, Conceptualization. **Michael Weldi:** Writing – review & editing, Resources, Project administration. **Gerald Trathnigg:** Writing – review & editing, Resources, Project administration. **Sebastian Billig:** Writing – review & editing, Writing – original draft, Visualization, Supervision, Investigation, Conceptualization.

## Funding

No external funding was received for this study or publication. SB was supported by the Clinician Scientist Program of the Faculty of Medicine, RWTH Aachen University.

## Declaration of competing interest

DP is a member of the Young ERC Committee and serves as Public Communications Officer for the Association for Emergency Medicine (AGN) in Styria. MW is National Course Director for Immediate Life Support (ILS) of the Austrian Resuscitation Council.

## Appendix A Supplementary material

Supplement 1 Expiratory Tidal volume, air leakage, and respiratory mechanical parameters of the Laerdal Resusci Anne Advanced SkillTrainer. This figure presents breath-by-breath measurements of expiratory tidal volume (VT_E_), air leakage, peak inspiratory pressure (PIP), plateau pressure (*P*_Plat_), and respiratory system compliance. Measurements were obtained using a facemask (FM), a first-generation laryngeal mask (LM), a second-generation LM (i-gel), a laryngeal tube (TL), and an endotracheal tube (ETT). Data are displayed as box plots (median, interquartile range, Tukey whiskers).
