# Supplementary material for: Ventilation characteristics of cardiopulmonary resuscitation manikins: a mixed-methods bench study
Source: Resusc Plus. 2026 Apr 7;29:101318. doi: 10.1016/j.resplu.2026.101318 (PMC13127201; doi:10.1016/j.resplu.2026.101318)

**Supplement 1**. Expiratory Tidal volume, air leakage, and respiratory mechanical parameters of the Laerdal Resusci Anne Advanced SkillTrainer.

This figure presents breath-by-breath measurements of expiratory tidal volume (VT_E_), air leakage, peak inspiratory pressure (PIP), plateau pressure (P_Plat_), and respiratory system compliance. Measurements were obtained using a facemask (FM), a first-generation laryngeal mask (LM), a second-generation LM (i-gel), a laryngeal tube (TL), and an endotracheal tube (ETT). Data are displayed as box plots (median, interquartile range, Tukey whiskers).


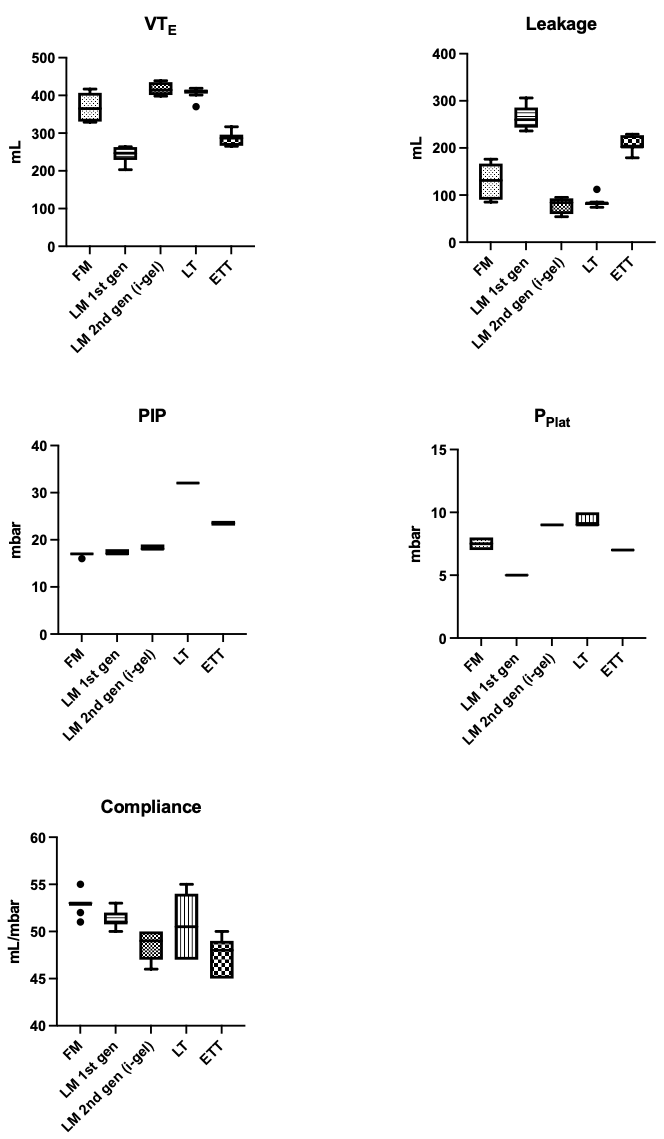

Supplement: Supplement 1 — Expiratory Tidal volume, air leakage, and respiratory mechanical parameters of the Laerdal Resusci Anne Advanced SkillTrainer. This figure presents breath-by-breath measurements of expiratory tidal volume (VTE), air leakage, peak inspiratory pressure (PIP), plateau pressure (PPlat), and respiratory system compliance. Measurements were obtained using a facemask (FM), a first-generation laryngeal mask (LM), a second-generation LM (i-gel), a laryngeal tube (TL), and an endotracheal tube (ETT). Data are displayed as box plots (median, interquartile range, Tukey whiskers). [file mmc1.docx]
